# Perceived benefits of the hepatitis C peer educators: a qualitative investigation

**DOI:** 10.1186/s12954-017-0192-8

**Published:** 2017-09-29

**Authors:** A. W. Batchelder, L. Cockerham-Colas, D. Peyser, S. P. Reynoso, I. Soloway, A. H. Litwin

**Affiliations:** 1 Montefiore Medical Center, Albert Einstein College of Medicine, 111 East 210th Street, Bronx, NY 10467 USA; 2 Behavioral Medicine, Department of Psychiatry, Massachusetts General Hospital, Harvard Medical School, One Bowdoin Square, 7th Floor, Boston, MA 02114 USA; 3 Rutgers, The State University of New Jersey, 607 Allison Road, Piscataway, NJ 08854 USA; 4 Division of Substance Abuse, Albert Einstein College of Medicine, 804 East 138th Street, Bronx, NY 10454 USA; 5 Port Morris Wellness Center, Montefiore Medical Center, Albert Einstein College of Medicine, Bronx, NY 10454 USA; 6 Einstein-Montefiore Division of General Internal Medicine, Department of Medicine, Albert Einstein College of Medicine, 3300 Kossuth Avenue, Bronx, NY 10467 USA; 7 Department of Psychiatry and Behavioral Science, Albert Einstein College of Medicine, 3300 Kossuth Avenue, Bronx, NY 10467 USA

**Keywords:** Hepatitis C, Peer, Peer educator, Methadone, Treatment

## Abstract

**Background:**

Although opioid-dependent patients are disproportionately impacted by hepatitis C (HCV), many do not receive treatment. In addition to HCV treatment-access barriers, substance-using patients may be reluctant to pursue treatment because of wariness of the medical system, lack of knowledge, or stigma related to HCV treatment. Implementation of a formal peer education program is one model of reducing provider- and patient-level barriers to HCV treatment, by enhancing mutual trust and reducing stigma.

**Methods:**

We used thematic qualitative analysis to explore how 30 HCV patients and peer educators perceived a HCV peer program within an established methadone maintenance program in the USA.

**Results:**

Participants unanimously described the program as beneficial. Participants described the peer educators’ normalization and dispelling of myths and fears around HCV treatment, and their exemplification of HCV treatment success, and reductions in perceived stigma. Peer educators described personal benefits.

**Conclusions:**

These findings indicate that HCV peer educators can enhance HCV treatment initiation and engagement within opioid substitution programs.

## Background

An estimated 3.5 million people are infected with hepatitis C (HCV) [1], with the majority of existing and new transmissions acquired from injection drug use [2]. In some methadone treatment programs, over 80% of opioid-dependent individuals have been exposed to HCV, with over 60% having chronic infection [3, 4]. HCV is curable in 75–90% of previously untreated patients who undergo treatment with triple therapy [5, 6], and now direct-acting antiviral drugs (e.g., sofosbuvir and simeprevir) have even greater success rates. Current national guidelines support the evaluation and treatment of past or current substance users [7–9], and evidence-based interventions exist to effectively deliver HCV treatment and maximize treatment outcomes [10].

However, many opioid-dependent patients still do not receive treatment [11–14]. Barriers to substance-using patients’ initiating HCV treatment include high rates of poverty, incarceration, homelessness, and discontinuity of medical care [15]. Additionally, medical providers’ may be hesitant to treat substance-using populations due to concerns about poor medication adherence, adverse side effects, co-morbid psychiatric illness, medical illness, and alcohol abuse, as well as the potential risk for re-infection [16, 17]. In addition to these barriers, substance-using patients may be reluctant to pursue HCV treatment because of wariness of the medical system, belief they do not need treatment, low levels of social support, or experienced or perceived stigma or shame related to approaching their provider about HCV treatment [15, 18, 19].

Peer education programs integrated into substance use treatment may reduce both provider- and patient-level barriers to HCV treatment by enhancing mutual trust, social support, and knowledge while reducing perceived stigma. We define peer educators as individuals who share common characteristics or experiences with those whom they are educating [20–22]. Evidence suggests that peer educators may be useful in addressing concerns related to HCV treatment initiation and engagement among individuals who use drugs [23, 24]. Previous studies have found that peer educators can help increase knowledge and engagement with HIV and HCV care [25–29] and that peer-to-peer interaction in a group setting can help reduce fears regarding medical procedures and treatment side effects [30]. While prior research has broadly examined the effects of peer educators, and some have explored the effects of peers on HCV treatment specifically in Australia and Canada [23, 31], this study is the first to our knowledge to focus on a qualitative analysis of patients’ perceptions, both peer educators and non-peer educators, of a HCV peer educator program within an established integrated HCV treatment and methadone maintenance program in a poor urban setting in the USA.

## Methods

### Setting

The Division of Substance Abuse (DoSA) at Albert Einstein College of Medicine (Einstein) is a network of three comprehensive treatment facilities for substance use disorders located in the Bronx, New York. Over 3500 adults receive pharmacotherapy services, including methadone maintenance and buprenorphine, intensive outpatient individual counseling, group counseling, and on-site primary medical care. The median age of patients at DoSA is 46 years (range, 28–72 years); 62% of the population is Hispanic, 20% is Black, and 18% is White; 61% are male. Sixty-five percent of the population exhibits HCV antibody positivity. Of those with a positive antibody, 75% have chronic HCV infection. Each year, over 200 patients initiate on-site HCV treatment.

The majority of DoSA’s patients visit the clinic daily to receive opiate agonist treatment, engage in ongoing medical care, and participate in various support and education groups. This daily attendance fosters regular patient-to-patient interactions, including frequent informal discussions between patients regarding their personal experiences, including treatment-related experiences. This culture provided the infrastructure for DoSA’s current HCV peer educator program.

The HCV peer educator program at DoSA enables trained peer educators to serve in a complementary, non-professional health role, providing physical and emotional support to patients via a formalized program [32]. HCV peer educators escort patients to off-site medical appointments, co-facilitate HCV education and treatment groups alongside health professionals, engage in clinic and community outreach and advocacy, and disseminate accurate HCV-related information to patients. Peer educators target individuals throughout all phases of the HCV disease spectrum, including prevention, pre-treatment, treatment, and post-treatment. Peer educators receive support, guidance, and feedback from designated HCV program staff and receive monetary stipends for the services they provide (specific details regarding the DoSA HCV peer program have been described elsewhere) [33]. New HCV peer educators are recruited annually, with HCV treatment-experienced patients being eligible to apply to the training. Candidates are required to participate in a structured 6-week training curriculum prior to being deemed official HCV peer educators.

### Participants

Between June 2011 and March 2013, we approached individuals who initiated HCV treatment. Individuals were eligible to participate in the study if they had initiated HCV treatment at one of DoSA’s three methadone maintenance clinics within the Einstein system. Participants were interviewed in a private room on site at their methadone clinic for 45–90 min by a member of the research team not involved in the implementation of the peer program. Interviews focused on participants’ experience initiating and undergoing HCV treatment, medication adherence, and substance use. Interviews were conducted with patients who interacted with peer educators within the HCV program and non-peer educators, as well as formerly trained peer educators, most of whom have stayed involved in the program. The Institutional Review Boards of Einstein approved this study.

Thirty adult patients (mean age = 51.3 (SD = 8.7)) were interviewed for this study. Two thirds of the sample was male. Twenty-two (73%) self-identified as Hispanic, three (13%) self-identified as African American or Black, and four (13%) self-identified as Caucasian or White. Thirteen (43%) of those interviewed had completed the HCV peer educator training, and the rest were non-peer educator participants. Seven (23%) of those interviewed were co-infected with HIV and HCV. All interviewees were HCV treatment experienced.

### Analysis

Our analytic approach followed Braun’s six steps of qualitative analysis [34]. Initially, two co-authors not involved in the implementation of the peer program listened to and read all interviews to identify general themes. Several themes related to perceptions of the peer program emerged through the open coding process, and a working coding tree was developed, which involved team members involved in the implementation of the peer program including the program director, coordinator, and clinicians. The full team then iteratively refined the coding structure. Open coding then continued, and discrepancies were brought to the team for discussion. After several iterations of the coding structure, transcripts were selectively coded by two co-authors, blinded to each other’s codes.

## Results

Our qualitative analysis identified overall positive perceptions of the peer program, including benefits experienced by interacting with peer educators and benefits experienced by the peer educators themselves. The non-peer educator participants described benefiting from experiencing the emotional support provided by the peer educators, having myths and fears related to HCV treatment dispelled, having their experiences normalized, and seeing examples of HCV treatment success. Together, these benefits were described as important in continued engagement and completion of HCV treatment. The peer educators described experiencing personal benefits from engaging in the peer educator role, including experiencing a feeling of expertise, a sense of accomplishment, and a sense of mission or purpose. These themes were described within the context of a pervasively positive HCV treatment experience.

### Patient benefits

The peer educators and non-peer educator participants described four main benefits from the HCV peer educator that contributed to sustained engagement and completion of treatment among those who had initiated treatment. These included emotional support informed by shared experiences, the dispelling of HCV-related myths and fears using accessible and relatable language, normalized HCV treatment-related experiences, and providing examples of HCV treatment success.

#### Emotional support

The participants described how peer educators provided advice, solidarity, and support or a listening ear when they or others were experiencing difficulty with HCV treatment. One peer educator described how she and other peer educators offered social support.

Somebody might have something that they’re going through. You help them work itout, give advice, or whatever we can do, and we spend a lot of time just being together. And that helped a lot. Being together. (Participant 17)

This emotional support was associated with an increased sense of strength during periods of vulnerability. One participant explained how peer support, including shared emotional experiences, enabled successful completion of the treatment regimen.

If it wasn’t [for] hear[ing] the peers talk about how they felt or talk about how I could beat the Hep C, I wouldn’t have the strength to keep going. (Participant 1*)*

#### Myths and fears dispelled

Many participants explained that misinformation about HCV was related to their initial reluctance to pursue treatment. The participants described the information provided by peer educators as equipping them with the knowledge and authority to dispel invalid information, thus enabling educated decisions about HCV treatment based on accurate information. One peer educator described his motivation to provide accurate information to keep people in HCV treatment.

Sometimes I would correct them because if they’re given wrong information they’re going to quit… You got to give them the right information. (Participant 3)

The peer educators described how experiential knowledge equipped them with the skills to calm anxious patients who were fearful to pursue treatment. One peer educator explained how her own treatment experience enabled her to reassure a patient who was apprehensive about the treatment.

She was so afraid of getting [HCV treatment] and I told her it’s nothing, trust me you’re going to feel better afterwards. (Participant 12)

The peer educators also described how speaking with or accompanying someone, as a person who had “been there” before, provided perspective to patients. Both the peer educators and non-peer educator participants reported this support reduced and at times eliminated patients’ fears regarding medical procedures. One peer educator described how simply accompanying patients to appointments, such as liver biopsies, mitigated fears about the procedures and increased the likelihood that the patient would attend the appointment.

…If somebody needs to go for a biopsy we’ll take ‘em. ‘Cause everybody’s afraid of that and they won’t do it if you go by yourself. (Participant 12)

##### Experiences are normalized

Both the peer educators and non-peer educator participants described the unique capacity of peer educators to offer comfort to those going through HCV treatment, given the shared lived experiences. The participants who had worked with a peer educator during their treatment reported feeling comforted knowing that the peer educator had undergone similar challenges, while on HCV treatment.

… It was good to talk to somebody that had basically the same problems that I had, that I could talk openly with. It wasn't like I was talking to my daughter or my cousin or my best friend; I was talking to another patient. And I felt that was very very helpful for my treatment. (Participant 21)

##### Living examples of treatment success

The participants described how interactions with peer educators increased their self-efficacy related to HCV treatment. Some explained how observing or learning about the treatment successes of peer educators exemplified that treatment success was possible. One non-peer educator participant explained how peer educators contributed to his decision to initiate HCV treatment.

[They] would give me advice and tell me you should try [the medication]…they told me they succeeded, that’s when I realized I’m going to make it. (Participant 1)

Several participants also described how peer educators’ treatment perseverance offered hope and inspiration, and conveyed that initiating and completing treatment is possible. One peer educator described how providing support by emphasizing shared lived experience helped keep patients engaged in treatment.

…You are helping somebody that wants to quit...they giving up…You tell them no, I’ve been there. You could do it. (Participant 3)

Another participant described how the peer educators’ shared experiences and perseverance motivated them to want to emulate their successful completion of HCV treatment.

The peers have an effect on me, you know, hearing them saying… the stages they were in… what the medication was doing to them…I wanted to see myself in them. (Participant 1)

Across the sample, both the peer educators and non-peer educator participants conveyed benefits provided to and experienced by those undergoing HCV treatment. These benefits included emotional support, providing accessible HCV treatment information, normalization of HCV treatment experiences, and providing examples of HCV treatment success. All of these benefits were enabled by the shared lived experience of the peer educators.

### HCV peer educator benefits

In addition to the benefits provided to those undergoing HCV treatment, the peer educators reported experiencing personal benefits as a result of this role. These benefits included being perceived as an expert and engaging in pro-social behavior.

#### Perceived as “experts”

All peer educators described positive and validating experiences associated with their role as a peer educator and their experienced and acquired HCV expertise. One peer educator shared how he felt in the role as a peer educator.

…I’m at the clinic- people there actually want to hear what I have to say, I’m excited, I’m telling you. (Participant 22)

The peer educators also reported that their newfound expertise was self-validating and created positive emotions and cognitions, including self-confidence. One participant explained how his role as a peer educator affected him.

I got confidence in myself…I talk to people. I tell them how I went through it… I tell people just stick to the medication. (Participant 1)

#### Pro-social behavior

The peer educators, and some patients who had not gone through the peer educator training, described social and meaning-based benefits of assisting other patients, including feeling engaged in pro-social behavior. One participant described how sharing his experience of treating his HCV helped another patient.

They ain’t taking no medication, no nothing. And by me telling them how I’m taking care of this... I’m pushing them to do the same thing that I’m doing…. I feel that I’m reaching out to somebody. (Participant 7)

The participants associated these meaningful pro-social engagements with positive feelings about themselves.

I feel great… If I reach one person to just go and just get tested…I feel like I accomplished something. Because that person now knows what their status is… (Participant 20)

## Discussion

In this study, we obtained perspectives from HCV treatment-experienced peer educators and non-peer educator patients on an established HCV peer educator program within a methadone maintenance program in the USA [33]. The participants overwhelmingly described HCV patient benefits related to engagement and completion of HCV treatment, including emotional support, clarification of myths and alleviation of fears related to HCV, normalization of HCV treatment experiences, and validation from shared lived experiences. The patients reported these benefits helped them stay motivated and engaged in HCV treatment. The participants who served as peer educators conveyed personal benefits from serving that role, including being perceived as HCV treatment experts. The peer educators also reported experiencing a sense of fulfillment and purpose related to engaging in pro-social behavior, which motivated several to aspire to continue to work toward engaging more people living with HCV in treatment.

Previous studies in Australia and Canada [23, 31, 35] have indicated that peer-based HCV programs show great promise in increasing HCV treatment uptake and adherence within existing HCV treatment programs. However, there is currently limited information regarding the utility of HCV peer education programs in the USA. Our findings provide qualitative evidence that HCV peer educators have the potential to address psychosocial barriers to HCV treatment initiation and engagement by providing accessible information about HCV, normalizing and addressing fears about treatment options, and minimizing stigma related to the healthcare system’s treatment of people who use substances, inject drugs, and have HCV [23]. HCV peer educators, who have experienced HCV treatment, may be uniquely situated to address these challenges through their ability to dispel myths, normalize treatment side effects, and provide factual information based on their personal experiences.

The perceived benefits of involvement in the HCV peer education program described here may be due to general benefits of support provided by those with similar treatment experiences that is distinct from the support provided by medical providers and family/friends without these experiences. The emerging literature describing HCV peer educators in other healthcare contexts indicates that peer programs may directly and indirectly affect patients undergoing HCV treatment [23, 36]. Involving peer educators in care for individuals with shared common psychological and health problems, including shared stigmatized identities, may be particularly helpful given the role HCV and substance use stigma may play as a barrier to HCV treatment initiation. Bandura famously found that when those we identify with accomplish what we aspire to accomplish, our self-efficacy increases [37]; this may account for the described impact the participants described for those initiating and engaging in HCV treatment. A formal peer educator program may also contribute to a clinical culture of empowerment, as the program demonstrates that patients can not only succeed in completing HCV treatment, as the peer educators have already done, but also have the capacity to advance to a clinically helpful role as a peer educator. The results from our study indicate that the involvement of peer education is perceived as empowering patients to remain motivated to complete HCV treatment. Our findings also indicate that the peer program was perceived to benefit peer educators themselves by increasing self-confidence and meaningful engagement in pro-social behavior.

It should be noted that the benefits of HCV peer educators may be experienced in the absence of a formalized peer educator program. Many of the same reported benefits in the sample were reported not only in relation to working with the trained peer educators, but also from interfacing with other *informal* peers going through HCV treatment. Results from our study were consistent with a HCV group treatment program described in Canada that included untrained peers, including sharing knowledge, lived experiences, allaying fears, and engaging in pro-social behavior [30]. Simply having a structure in place that allows treatment-experienced patients to interact with non-treatment experienced patients may produce similar benefits.

However, the perceived benefits of a formal peer education program should not be underestimated. Based on their experience in Australia, Treloar et al. and Woolhouse et al. state that peers are not as equipped to effectively support and provide guidance to patients considering, initiating, and engaging in HCV treatment without a supportive structure, physical space, and support from authorities [23, 30]. Other programs in Australia have found a peer-based integrated model of HCV care to be acceptable and feasible for injection drug users [38]. Formalized peer education programs inherently provide this supportive framework that facilitates the benefits found in our research.

The findings of our study may be due in part to the unique clinic setting, which provided a framework for our peer educators to succeed. This involved comprehensive on-site HCV treatment including a multidisciplinary HCV treatment team of internal medicine specialists, nurses, substance abuse counseling staff, and a psychiatrist [8]. The HCV treatment program includes the option to attend regular HCV support groups and the formal peer educator program [39]. The participants in this program have reported psychological and behavioral transformation [40]. In this atypical integrated HCV care model, there is a 13-year tradition of peer educators working with providers to inform HCV care and advocacy. This model and clinic culture was developed to empower patients to become peer educators and advocate and iteratively contribute to improving the system of care. This type of integrated clinic setting may enable and perpetuate the type of peer support described by the participants.

## Conclusions

With the advent of newer medications with increasingly higher cure rates, it is likely that growing numbers of patients will be encouraged and motivated to pursue HCV treatment. The increased patient volume of opioid-dependent people living with HCV, coupled with the high costs of HCV treatment, will likely create an atmosphere that requires the limited HCV treatment-related resources to be used effectively. Our study reveals that peer educators can play an important role in the initiation, engagement, and successful completion of HCV treatment among opioid-dependent people living with chronic HCV. HCV peer education programs may offer a cost-effective and appropriate solution to current psychosocial barriers to HCV treatment among this population, including inaccurate beliefs about treatment needs, low social support, wariness of the medical system, and experienced or perceived stigma or shame related to HCV and substance use [33]. While barriers remain to accessing HCV treatment in the USA and elsewhere, HCV peer educator programs have the potential to significantly reduce psychosocial barriers to HCV treatment initiation rates and improve outcomes.

## Abbreviations

DoSA: Division of Substance Abuse
HCV: Hepatitis C

## References

[1] Edlin BR, Eckhardt BJ, Shu MA, Holmberg SD, Swan T (2015). Toward a more accurate estimate of the prevalence of hepatitis C in the United States. Hepatology.

[2] Centers for Disease Control and Prevention. Available at: http://www.cdc.gov/hepatitis/hcv/hcvfaq.htm. Accessed 20 Sept 2017.

[3] McCarthy JJ, Flynn N (2001). Hepatitis C in methadone maintenance patients: prevalence and public policy implications. J Addict Dis.

[4] Stein MD, Maksad J, Clarke J (2001). Hepatitis C disease among injection drug users: knowledge, perceived risk and willingness to receive treatment. Drug Alcohol Depend.

[5] Jacobson IM, McHutchison JG, Dusheiko G (2011). Telaprevir for previously untreated chronic hepatitis C virus infection. N Engl J Med.

[6] Lawitz E, Sulkowski MS, Ghalib R (2014). Simeprevir plus sofosbuvir, with or without ribavirin, to treat chronic infection with hepatitis C virus genotype 1 in non-responders to pegylated interferon and ribavirin and treatment-naive patients: the COSMOS randomised study. Lancet.

[7] Schulte B, Schütt S, Brack J (2010). Successful treatment of chronic hepatitis C virus infection in severely opioid-dependent patients under heroin maintenance. Drug Alcohol Depend.

[8] Litwin AH, Harris KA, Nahvi S (2009). Successful treatment of chronic hepatitis C with pegylated interferon in combination with ribavirin in a methadone maintenance treatment program. J Subst Abus Treat.

[9] Health NIO (2002). National Institutes of Health Consensus Development Conference Statement: management of hepatitis C: 2002—June 10–12, 2002. Hepatology (Baltimore, Md).

[10] Meyer JP, Moghimi Y, Marcus R, Lim JK, Litwin AH, Altice FL (2015). Evidence-based interventions to enhance assessment, treatment, and adherence in the chronic hepatitis C care continuum. International Journal of Drug Policy..

[11] Hellard M, Sacks-Davis R, Gold J (2009). Hepatitis C treatment for injection drug users: a review of the available evidence. Clin Infect Dis.

[12] Strathdee SA, Latka M, Campbell J (2005). Factors associated with interest in initiating treatment for hepatitis C virus (HCV) infection among young HCV-infected injection drug users. Clin Infect Dis.

[13] Mehta SH, Genberg BL, Astemborski J (2008). Limited uptake of hepatitis C treatment among injection drug users. J Community Health.

[14] Wolfe D, Luhmann N, Harris M (2015). Human rights and access to hepatitis C treatment for people who inject drugs. International Journal of Drug Policy..

[15] Edlin BR, Kresina TF, Raymond DB (2005). Overcoming barriers to prevention, care, and treatment of hepatitis C in illicit drug users. Clin Infect Dis.

[16] Edlin BR, Seal KH, Lorvick J (2001). Is it justifiable to withhold treatment for hepatitis C from illicit-drug users?. N Engl J Med.

[17] Davis GL, Rodrigue JR (2001). Treatment of chronic hepatitis C in active drug users. N Engl J Med.

[18] Chen E, North C, Fatunde O (2013). Knowledge and attitudes about hepatitis C virus (HCV) infection and its treatment in HCV mono-infected and HCV/HIV co-infected adults. J Viral Hepat.

[19] Munoz-Plaza CE, Strauss S, Astone-Twerell J (2008). Exploring drug users’ attitudes and decisions regarding hepatitis C (HCV) treatment in the US. International Journal of Drug Policy..

[20] Shiner M (1999). Defining peer education. J Adolesc.

[21] Simoni JM, Franks JC, Lehavot K, Yard SS (2011). Peer interventions to promote health: conceptual considerations. Am J Orthopsychiatry.

[22] Simoni JM, Nelson KM, Franks JC, Yard SS, Lehavot K (2011). Are peer interventions for HIV efficacious? A systematic review. AIDS Behav.

[23] Treloar C, Rance J, Bath N (2015). Evaluation of two community-controlled peer support services for assessment and treatment of hepatitis C virus infection in opioid substitution treatment clinics: the ETHOS study, Australia. International Journal of Drug Policy.

[24] Rance J, Treloar C. Integrating treatment: key findings from a qualitative evaluation of the Enhancing Treatment of Hepatitis C in Opiate Substitution Settings (ETHOS) study: National Centre in HIV Social Research; 2012. https://csrh.arts.unsw.edu.au/media/CSRHFile/2_Integrating_treatment.pdf.

[25] Broadhead RS, Heckathorn DD, Weakliem DL, Anthony DL, Madray H, Mills RJ, Hughes J (1998). Harnessing peer networks as an instrument for AIDS prevention: results from a peer-driven intervention. Public Health Rep.

[26] Cottler LB, Compton WM, Abdallah AB (1998). Peer-delivered interventions reduce HIV risk behaviors among out-of-treatment drug abusers. Public Health Rep.

[27] Broadhead RS, Heckathorn DD, Altice FL (2002). Increasing drug users’ adherence to HIV treatment: results of a peer-driven intervention feasibility study. Soc Sci Med.

[28] Garfein RS, Golub ET, Greenberg AE (2007). A peer-education intervention to reduce injection risk behaviors for HIV and hepatitis C virus infection in young injection drug users. AIDS.

[29] Medley A, Kennedy C, O’Reilly K, Sweat M (2009). Effectiveness of peer education interventions for HIV prevention in developing countries: a systematic review and meta-analysis. AIDS education and prevention: official publication of the International Society for AIDS Education.

[30] Woolhouse S, Cooper E, Pickard A (2013). “It gives me a sense of belonging”: providing integrated health care and treatment to people with HCV engaged in a psycho-educational support group. International Journal of Drug Policy..

[31] Mason K, Dodd Z, Sockalingam S (2015). Beyond viral response: a prospective evaluation of a community-based, multi-disciplinary, peer-driven model of HCV treatment and support. International Journal of Drug Policy.

[32] Litwin AH, Soloway I, Gourevitch MN. Integrating services for injection drug users infected with hepatitis C virus with methadone maintenance treatment: challenges and opportunities. Clin Infect Dis. 2005;40(Supplement 5):S339-S45. 10.1086/427450 15768345

[33] Roose RJ, Cockerham-Colas L, Soloway I, Batchelder A, Litwin AH (2014). Reducing barriers to hepatitis C treatment among drug users: an integrated hepatitis C peer education and support program. J Health Care Poor Underserved.

[34] Braun V, Clarke V (2006). Using thematic analysis in psychology. Qual Res Psychol..

[35] Keats J, Micallef M, Grebely J (2015). Assessment and delivery of treatment for hepatitis C virus infection in an opioid substitution treatment clinic with integrated peer-based support in Newcastle, Australia. International Journal of Drug Policy.

[36] Rance J, Treloar C (2014). ‘Not just methadone Tracy’: transformations in service-user identity following the introduction of hepatitis C treatment into Australian opiate substitution settings. Addiction.

[37] Bandura A (1988). Self-efficacy conception of anxiety. Anxiety research.

[38] Norman J, Walsh N, Mugavin J, Stoové M, Kelsall J, Austin K, Lintzeris N (2008). The acceptability and feasibility of peer worker support role in community based HCV treatment for injecting drug users. Harm Reduction Journal.

[39] Stein MR, Soloway IJ, Jefferson KS, Roose RJ, Arnsten JH, Litwin AH (2012). Concurrent group treatment for hepatitis C: implementation and outcomes in a methadone maintenance treatment program. J Subst Abus Treat.

[40] Batchelder A, Peyser D, Nahvi S, Arnsten J, Litwin A (2015). “Hepatitis C treatment turned me around:” psychological and behavioral transformation related to hepatitis C treatment. Drug Alcohol Depend.

